# Ethnic differences in pain, function, and catastrophizing in South Florida adults with knee osteoarthritis

**DOI:** 10.1371/journal.pone.0329741

**Published:** 2025-08-04

**Authors:** Daniel Quintero, Jacob Jahn, Jean Jose, Eric Kholodovsky, Levi M. Travis, Joseph P. Costello II, Olivia Perez, Alberto J. Caban-Martinez, Thomas M. Best

**Affiliations:** 1 Department of Orthopedics, Division of Sports Medicine, UHealth Sports Medicine Institute, Miami, Florida, United States of America,; 2 Department of Radiology, University of Miami Miller School of Medicine, Miami, Florida, United States of America; 3 Department of Public Health Sciences, University of Miami Miller School of Medicine, Miami, Florida, United States of America; The Affiliated Changzhou No 2 People's Hospital of Nanjing Medical University, CHINA

## Abstract

**Objective:**

Ethnicity is associated with varying reporting of pain, coping mechanisms, and disease severity in patients with knee osteoarthritis (KOA). However, few studies have evaluated its importance in ethnicity, particularly the Hispanic population. This study compares pain intensity (VAS), function (WOMAC), and pain catastrophizing (PCS) between Hispanic **(HP)** and non-Hispanic White patients **(NHWP)** stratified by socio-economic status (SES) and osteoarthritis radiographic K-L grade.

**Methods:**

A cross-sectional study of patients from a tertiary care clinic between July 2021 and December 2022 was performed. Patients with knee pain, radiographs, and doctor-diagnosis of KOA completed questionnaires in English or Spanish. Descriptive statistics characterized demographic differences between **NHWP** and **HP** in VAS, WOMAC, and PCS. Two one-way analyses of variance evaluated the effect of both ethnicity and sex, with subgroup analyses stratifying by K-L grade. Multivariate general linear models assessed primary outcomes while controlling for confounders.

**Results:**

A total of 195 subjects (**HP** = 145, **NHWP** = 50) were included. **HP** exhibited higher VAS, PCS, and WOMAC scores compared to **NHWP**. PCS was higher in **HP** (p = 0.004, mean = 8.89) than **NHWP** (mean = 4.58), as was **VAS** (p < 0.001, mean = 4.28 vs. 2.74) and WOMAC (p = 0.029, mean = 27.86 vs. 21.58). These differences remained when controlled for NSES and K-L grade. Stratifying by sex and comparing primary outcomes between **HP** and **NHWP**, male **HP** had greater VAS (p = .021, mean = 3.83 vs. 2.42) and PCS (p = .008, mean = 8.83 vs. 3.35), while female **HP** had greater **VAS** (p = .019, mean = 4.62 vs. 3.08) and nonsignificantly greater PCS (p = .164, mean = 8.94 vs. 5.92).

**Conclusion:**

**HP** with KOA reported greater pain severity, functional limitation, and PCS.

compared with **NHWP**, even after adjusting for NSES and K-L grade. Our findings expand on previous reports by including a larger number of HP and analyzing the role of sex, impact of socioeconomic status, and influence of radiographic severity on patient symptoms.

## Introduction

Osteoarthritis (OA) is the most common form of arthritis worldwide, with a higher incidence in high-income countries and older individuals. In the United States alone, over 32.5 million individuals have been diagnosed with OA, 3 million of which are Hispanic, a minoritized ethnicity [1–4]. The burden of OA in the Hispanic community is significant, contributing to increased disruption to daily activities, health disparities, and prevalence. Studies have shown that Hispanic individuals (HP) with OA often experience greater pain severity and disability compared to non-Hispanic White patients (**NHWP**), highlighting the importance of addressing OA in this population [5,6]. Additionally, HP are less likely to receive appropriate OA management, which can negatively influence health outcomes [7]. Knee OA (KOA) is the leading cause of lower extremity disability, and as of 2010, was ranked the 11th highest contributor to global disability [8]. Accordingly, studies including diverse populations and larger sample sizes are needed to more fully understand the total burden of OA to better inform unmet needs of both the patient and the medical community serving them.

The diagnosis of OA is typically based on clinical assessment and confirmed by routine plain radiographs [6]. Patients typically report activity-related joint pain that is associated with morning stiffness of less than 30 minutes duration [9,10]. However, this presentation is not consistent across all patient populations. Like other chronic diseases, the presentation and subsequent morbidity of KOA demonstrates considerable variability across different races and, more recently, ethnicities [11–15]. For example, the Johnston County OA project demonstrated statistically significant differences in both pain severity and radiographic severity (Kellgren and Lawrence [K-L]) scores between African Americans and Caucasians with symptomatic KOA at initial presentation [16,17]. Other investigators have corroborated this finding by reporting greater pain severity among African Americans compared to **NHWP** with hip and knee OA [18–24]. Both studies utilized race, an observable characteristic with social implications, as the independent variable between groups. Recently, Hoffman et al. denoted that there are no biological differences in pain experience based on race alone yet there are racial biases in the treatment and assessment of pain [25].

Ethnicity, on the other hand, may or may not carry a different race; however, the term attempts to capture the culture of people in a given geographic region, including their language, heritage, religion and customs [18]. Hollingshead et al. discussed how ethnicity is involved in the processing and reporting of pain in individuals with one or more chronic diseases [26,27]. Moreover, the investigations by Hollingshead observed that Hispanic persons **(HP)** report greater pain sensitivity and less pain tolerance than **NHWP** [26]. Furthermore, a study by Wang included four different ethnicities (East Asian, African American, Hispanic and Caucasian) and found differences in pain sensitivity between ethnicity after capsaicin administration [28,29]. Finally, Levy et al. highlighted differences in symptom severity on presentation for a well-known chronic illness, alcoholic liver disease, between Caucasians and Hispanics [30].

Although current data indicating an association between pain experience, disease severity, and ethnicity are limited, there are findings specific in Hispanics with chronic pain and arthritis regarding pain intensity and coping mechanisms. Studies utilizing population-based data from the third National Health and Nutrition Examination Survey (NHANES-III) demonstrated that **HP** with self- reported arthritis are twice as likely to self-report symptoms of activity limitation when compared to **NHWP** [2]. **HPs** with an assigned diagnosis of arthritis also tend to report greater pain scores as measured by the Visual Analog Scale (VAS) [18]. Furthermore, when compared to **NHWP**, **HPs** have a greater propensity to utilize pain-catastrophizing coping mechanisms to address any physical manifestation of chronic pain [18,27]. The psychosocial aspect of arthritic pain and how implications in socioeconomic, psychological, and environmental factors have a substantial influence on an individual’s perception of their self-efficacy and quality of life have been highlighted [29–31].

One maladaptive strategy utilized more commonly in Hispanics for coping with chronic pain is pain catastrophizing (PCS). Pain catastrophizing is the tendency to develop a belief that the pain will not resolve (helplessness), a constant repetitive dwelling on the negative aspects of the pain (rumination) and exaggerating/ falsely intensifying the perceived pain stimulus (magnification) [32]. Greater pain catastrophizing is associated with greater pain and functional limitation [33]. Additionally, greater pain catastrophizing has been independently associated with lower socio-economic status [12,34–37]. Green et al. expanded on this claim and underlined that neighborhood socioeconomic status (NSES) mediated chronic pain experience in younger White and Black individuals [38]. They argued that NSES mediated the pain experience differences identified between races. Therefore, to explore if differences in pain experiences exists between ethnicity, NSES or a surrogate measure may be noteworthy [36,38].

As in a number of chronic diseases, the elements contributing to OA symptomology are multifactorial; therefore, it is important to consider whether ethnic differences in pain sensitivity or PCS might be moderated by biological sex. To investigate this further, our study conducts specific demographically based analyses, such as breaking down participants by sex, to investigate ethnic differences in pain outcome measures like PCS. For instance, previous research has shown that sex differences in pain perception and coping strategies are notable, with women often reporting higher pain sensitivity and greater pain catastrophizing compared to men [39–41]. Additionally, the intersectionality of sex and ethnicity may further elucidate these disparities in pain experiences, as cultural norms and expectations related to sex and ethnicity can also impact pain reporting and management [42,43]. Therefore, it has been hypothesized that sex may influence the relationship between ethnicity and pain outcomes, with Hispanic women possibly reporting higher pain sensitivity and greater pain catastrophizing compared to their male counterparts and **NHWP** women [44,45].

The purpose of the present study was to determine if there are differences in pain severity, functional limitation, and pain catastrophizing between **HP** and **NHWP** with KOA after adjusting for the possible confounders including socioeconomic status (SES) and radiographic disease severity. Investigating a convenience sample of patients from southern Florida, a region somewhat unique with its high prevalence of **HP**, our hypothesis was that there are differences in reporting of pain severity, functional limitations, and pain catastrophizing between the two ethnicities that will persist after controlling for covariates including NSES and KL grade.

## Materials and methods

### Study population and data source

All procedures were approved by the University of Miami Institutional Review Board (IRB). 195 study participants were recruited between July 1st, 2021 and December 22^nd^, 2022 from our tertiary care medical center in Miami-Dade County, Florida via convenient sampling contingent on investigator (DQ, JPC) availability and patient willingness. Patient eligibility was limited to those eligible to be seen at our clinic (valid insurance coverage, age greater than 18) presenting for the first time to our clinic with knee pain and/or stiffness and subsequently diagnosed with KOA by history, physical exam, and knee radiographs (i.e., AP, lateral and skyline Merchant views). Clinical OA was determined by a sports medicine attending (TMB) during the encounter. OA grade was determined by a blinded musculoskeletal radiologist (J Jose) using the KL grading system (4). Location of OA (tibiofemoral and/or patellofemoral) was also noted. Following written patient consent, one of four interviewers (DQ, SH, JPC, EK) administered a series of standard questionnaires in either English or Spanish depending on patient preference. All required data were collected using REDCap, an electronic data capture tool hosted at the University of Miami. The data were handled by a single investigator (DQ), de-identified by random sequence software using a university- approved computer and stored on an encrypted hard drive. Written consent was obtained for each patient and was provided in both English and Spanish. The study research protocol was reviewed and approved by the University Institutional Review Board (#20190389) at the University of Miami.

### Survey instrument and study measures

We administered a 61-item study questionnaire during the clinical encounter to collect information about participants’ sociodemographic characteristics, including age, sex, occupation, smoking status, race, and body mass index. As part of the questionnaire, we issued the Charles comorbidity index (CCI) to quantify participants’ chronic comorbid conditions. Nineteen different conditions were assessed among study participants including, myocardial infarction, congestive heart failure, peripheral vascular disease, cerebrovascular disease (except hemiplegia), dementia, chronic pulmonary disease, connective tissue disease, ulcer disease, mild liver disease, diabetes (without complications), diabetes with end organ damage, hemiplegia, moderate or severe renal disease, solid tumor (metastatic and non-metastatic), leukemia, lymphoma, multiple myeloma, moderate or severe liver disease, and HIV infection. These conditions were included as they represent a wide spectrum of chronic diseases that can impact overall health and function, providing a comprehensive measure of the existing comorbidity burden. The presence of individual diseases was quantified using a weighted point system and added to produce a single value per participant [36].

Patient pain scores were quantified at the time of presentation to the clinic using a scale from 0 to 10 (visual analog scale – VAS). Before answering, the patient was told that 0 represented being pain-free and 10 represented the worse pain imaginable. We also administered a standardized pain catastrophizing scale (PCS) questionnaire. This scale was developed to assess three components of catastrophizing: rumination, magnification, and helplessness [37]. The scale included 13 validated questions that required responses on a point scale of 0–4, and the scores were summed to produce a single value representing the total pain catastrophizing exhibited by each participant, with higher PCS indicating greater pain catastrophizing [37]. Participant functional limitation was assessed using the Western Ontario and McMaster Universities Osteoarthritis Index (WOMAC) [11], a validated tool specifically designed to evaluate pain, stiffness, and physical functionality in daily life in individuals with OA.

### Spanish validation and testing administration

Investigators (DQ and JPC) are qualified per IRB requirements to gather consent and subsequently administered the 61-item questionnaire in Spanish to study participants. All questionnaires CCI, PCS, VAS, and WOMAC were previously validated in Spanish [46–50].

### Geocoding socioeconomic status

We replicated the approach used by *Feldman et al*. and utilized the Geographic Information System (GIS) to geocode individual home addresses [34]. For each patient’s individual address, we obtained a Federal Information Processing Standard (FIPS) code and linked it to U.S. Census and American Community Survey data at the block group level. The block group is the smallest geographic unit for which the variables are published. For each block, we collected the Neighborhood Socioeconomic Status (NSES) Index, a previously validated measure for socioeconomic status [51]. This measure includes unemployment (percentage of persons ≥16 years of age in the labor force who are unemployed and actively seeking work), income (percentage of persons below the federally defined poverty line and median household income), wealth (median value of owner-occupied homes), education (percentage of persons aged >25 with less than a 12th-grade education and percentage of persons aged >25 years with at least four years of college) and crowding (percentage of households containing ≥1 person per room). SES index scores were reported on a scale of 0–100. NSES index values for each identified block pertaining to a single ethnicity were averaged and reported as a mean and standard deviation.

### Data analysis

Continuous values were reported as the mean and standard deviation for each sample. For descriptive data defined by dichotomous selection, the total percentage within a category was reported.

### Comparisons

Sample means were compared between groups via Student’s T-test for comparison of means. For comparisons of normally distributed continuous variables between two independent groups, independent samples t-tests were used. Comparisons of the study parameter that failed F-testing required a Mann-Whitney test for averages of unequally distributed sample values. Statistical comparisons of survey data were performed with GraphPad Prism Version 9.4.1 software (GraphPad Software, San Diego, California USA,).

### Sub-group analysis

Descriptive statistics were generated with means and standard deviations for continuous variables and frequencies with proportions for categorical variables. The three primary dependent variables evaluated included VAS (1–10 scale), PCS, and WOMAC. K-L grade was evaluated as both an independent variable, and as a secondary dependent variable given specific secondary independent variables (Age, BMI, etc.) may influence disease severity radiographically.

Stratified analyses were performed based on a priori hypotheses and supported by observed trends in interaction terms. Although formal interaction testing was not always statistically significant, stratification helped to explore clinically meaningful trends by sex and radiographic severity. For comparisons of outcomes between ethnic groups within each K-L grade stratum, independent samples t-tests were used.

### ANOVA analysis

One-way analyses of variance (ANOVA) were performed to evaluate the effect of both dichotomous independent variables: Ethnicity (**NHWP** or **HP**) and Sex (M or F). WOMAC, PCS, VAS, and K-L grade were analyzed as dependent variables. One-way ANOVAs were also performed comparing WOMAC, PCS, and VAS of ethnicities while stratifying by K-L grade and Sex. A one-way ANOVA was performed comparing the effect of K-L grade on WOMAC, PCS, and VAS, while stratifying by Ethnicity. One-way ANOVAs were also performed to compare means of Age, Height, CCI, and NSES between **NHWP** and **HP**.

While mean K-L grade values were reported descriptively, comparisons by K-L grade category were conducted using non-parametric tests and ANOVA treating K-L as categorical. Any analysis involving K-L as a covariate in multivariable models treated it as ordinal with fixed levels

### Linear regressions

Linear regressions were performed to evaluate the effects of all continuous or scale independent variables (NSES, CCI, Height, BMI, and Age) on each of the three primary dependent variables, as well as K-L grade. The relationships between continuous covariates such as BMI and outcome variables (PCS, WOMAC, and VAS) were evaluated using linear regression. Observed cumulative probability was plotted against expected cumulative probability. Standardized residuals were plotted in histogram form to validate sample distribution, and standardized predicted residuals were plotted against actual standardized residuals to check validity of each regression model.

### Multivariate analysis

Multivariate general linear models were computed for primary independent variables and their interaction between each other and various secondary independent variables, including key continuous variables. Selected variables were based on the results of one-way ANOVAs and linear regressions, which demonstrated isolated effects of particular independent variables and therefore had to be cross-analyzed with primary independent variables to see interaction and confounding effects. Variables found to have potential confounding effects on primary independent variables, such as socioeconomic status and K-L grade, were then tested in multivariate general linear models as covariates both separately and in the same model. Post-hoc Tukey and Bonferroni tests were computed for analyses that contained 3 or more subgroups, which for this sample included only K-L grade. Similarly, univariate general linear models were computed for primary independent variables and secondary independent variables found to have statistically significant effects on VAS, PCS, or WOMAC.

Graphs were created to demonstrate both the effects of primary independent variables, as well as primary independent variables stratified by other variables shown to have significant or non-significant effect on VAS, PCS, or WOMAC. Scatterplots were generated to demonstrate correlation and R^2^ values of linear regressions. Mean and median plots were generated, depending on the scale of the dependent variable and the vulnerability of the variable to have significant outliers, such as PCS. All graphs were generated using IBM SPSS statistics version 29.0.2.0 or Microsoft Excel version 16.83.

All statistical analysis was conducted using IBM SPSS Statistics version 29.0.2.0. Statistical significance was considered alpha values <0.05 and within a 95% confidence interval.

## Results

### Outcomes by ethnicity

**Fig 1:**
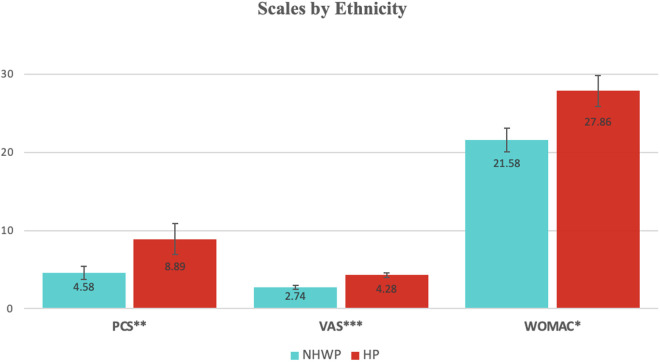
Comparison of outcomes PCS, VAS, WOMAC between HP and NHWP. Values with asterisk (*) indicate significance. p values for (*)= < .05, (**)=<.01, (***)=<.001.

VAS, PCS, and WOMAC were greater in **HP** when compared to **NHWP** (**Table 1**, **Fig 1**). The mean PCS score was greater in **HP** (p = 0.004, t(193) = −2.919, d = −.479) at 8.89 (STE = 0.814, CI = 95% [7.28–10.50]) while **NHWP** had a mean PCS of 4.58 (STE = 0.862, CI = 95% [2.85–6.31]). For VAS, the mean was again greater in **HP** (p < 0.001, t(193) = −3.474, d = −.570) at 4.28 (STE = 0.236, CI = 95% [3.81–4.74]) while **NHWP** had a mean VAS rating of 2.74 (STE = 0.313, CI = 95% [2.11–3.37]). The mean WOMAC in **HP** was greater than **NHWP** (p = 0.029, t(193) = −2.195, d = −.360) with an average of 27.86 (STE = 1.536, CI = 95% [24.82–30.89]) in **HP** and 21.58 (STE = 1.960, CI = 95% [17.64–25.52]) in **NHWP**. K-L grade demonstrated no difference between **HP** and **NHWP** (p = 0.581, t(193) = 0.553, d = .091) with mean grades of 1.66 (STE = 0.096, CI = 95% [17.64–25.52]) and 1.76 (STE = 1.960, CI = 95% [17.64–25.52]) respectively. When stratifying by ethnicity, K-L grade showed no significant effect on VAS, PCS, or WOMAC in **NHWP**. However, increased K-L grade showed a significant increase in VAS, PCS, and WOMAC in **HP** (**Table 2**). Population sizes for **NWHP** at each K-L grade ranged from n = 5 to n = 17, while in **HP** they ranged from n = 14 to n = 52 (**Table 3**).

**Table 1:** Comparison of VAS, PCS, and WOMAC in HP and NHWP populations, as well as comparisons of demographic information such as Age, Height, CCI, and NSES. Bolded values indicate significance.

	Measure	Age		Height		CCI		VAS		PCS		WOMAC		Kellgren and Lawrence Grade		SES	
		NHWP	HP	NHWP	HP	NHWP	HP	NHWP	HP	NHWP	HP	NHWP	HP	NHWP	HP	NHWP	HP
**N**	Valid	50	145	50	145	50	145	50	145	50	145	50	145	50	145	50	145
Mean		60.18	56.18	67.72	65.92	1.94	1.74	2.74	4.28	4.58	8.89	21.58	27.86	1.76	1.66	63.77	55.2
Median		59.5	55	67	65	2	1	3	5	3	6	19.5	26	2	1	67.75	53.9
Std. Dev.		10.753	11.083	4.081	3.944	1.544	1.654	2.211	2.842	6.098	9.798	13.862	18.492	1.17	1.151	16.4723	13.3016
Range		41	51	14	19	6	8	10	10	28	42	59	85	4	5	61.6	54.3
**Percentile**	25 Percentile	50.75	48.5	64	62.5	1	1	1	2	1	3	11	13	1	1	51.15	45.65
	50 Percentile	59.5	55	67	65	2	1	3	5	3	6	19.5	26	2	1	67.75	53.9
	75 Percentile	69.25	65	72	69	3	3	4	6	6	12	32.75	40.5	2	2	78.25	65.25

**Table 2:** Comparison of VAS, PCS, and WOMAC by K-L grade, stratified by Ethnicity.

Ethnicity		N	Mean	Std. Deviation	Std. Error	95% CI Lower Bound	95% CI Upper Bound
**NHWP**							
**VAS**	0	6	1.83	1.472	0.601	0.29	3.38
	1	17	2.18	2.378	0.577	0.95	3.4
	2	16	3.38	2.062	0.515	2.28	4.47
	3	5	2.2	1.924	0.86	−0.19	4.59
	4	6	4	2.53	1.033	1.35	6.65
	Total	50	2.74	2.211	0.313	2.11	3.37
**PCS**	0	6	1.5	1.643	0.671	−0.22	3.22
	1	17	7.24	8.02	1.945	3.11	11.36
	2	16	2.44	3.521	0.88	0.56	4.31
	3	5	4	6.364	2.846	−3.9	11.9
	4	6	6.33	5.68	2.319	0.37	12.29
	Total	50	4.58	6.098	0.862	2.85	6.31
**WOMAC**	0	6	10.5	9.975	4.072	0.03	20.97
	1	17	22.35	11.942	2.896	16.21	28.49
	2	16	24.75	16.482	4.121	15.97	33.53
	3	5	24.4	9.45	4.226	12.67	36.13
	4	6	19.67	15.718	6.417	3.17	36.16
	Total	50	21.58	13.862	1.96	17.64	25.52
**HP**							
**VAS**	0	21	2.9	2.879	0.628	1.59	4.22
	1	52	3.92	2.535	0.352	3.22	4.63
	2	42	4.26	2.812	0.434	3.39	5.14
	3	16	6.31	2.442	0.61	5.01	7.61
	4	14	5.36	3.128	0.836	3.55	7.16
	Total	145	4.28	2.842	0.236	3.81	4.74
**PCS**	0	21	5.71	7.128	1.556	2.47	8.95
	1	52	8.02	0.982	1.26	5.49	10.55
	2	42	4.86	3.136	0.484	3.86	5.86
	3	16	12.56	12.796	3.199	5.65	22.32
	4	14	10.79	11.477	3.067	4.16	17.41
	Total	145	8.89	9.798	0.814	7.28	10.5
**WOMAC**	0	21	20.81	16.833	3.673	13.15	28.47
	1	52	22.94	16.281	2.258	18.41	27.48
	2	42	30.02	18.122	2.796	24.38	35.67
	3	16	40.94	20.349	5.087	30.9	51.78
	4	14	25.21	18.377	4.912	16.2	45.82
	Total	145	27.86	18.492	1.536	24.82	30.89

**Table 3:** Comparison of outcomes VAS, PCS, and WOMAC between HP and NHWP, stratified by K-L grade.

							95% Confidence Interval for Mean	
Kellgren and Lawrence Grade	Measure	Ethnicity	N	Mean	Std. Deviation	Std. Error	Lower Bound	Upper Bound
0	VAS	NHWP	6	1.83	1.472	0.601	0.29	3.38
		HP	21	2.9	2.879	0.628	1.59	4.22
		Total	27	2.67	2.646	0.509	1.62	3.71
0	PCS	NHWP	6	1.5	1.643	0.671	−0.22	3.22
		HP	21	5.71	7.128	1.556	2.47	8.96
		Total	27	4.78	6.542	1.259	2.19	7.37
0	WOMAC	NHWP	6	10.5	9.975	4.072	0.03	20.97
		HP	21	20.81	16.833	3.673	13.15	28.47
		Total	27	18.52	16.006	3.08	12.19	24.85
1	VAS	NHWP	17	**2.18**	2.378	0.577	0.95	3.4
		HP	52	**3.92**	2.535	0.352	3.22	4.63
		Total	69	3.49	2.593	0.312	2.87	4.12
1	PCS	NHWP	17	7.24	8.02	1.945	3.11	11.36
		HP	52	8.02	9.082	1.26	5.49	10.55
		Total	69	7.83	8.782	1.057	5.72	9.94
1	WOMAC	NHWP	17	22.35	11.942	2.896	16.21	28.49
		HP	52	22.94	16.281	2.258	18.41	27.48
		Total	69	22.8	15.246	1.835	19.13	26.46
2	VAS	NHWP	16	3.38	2.062	0.515	2.28	4.47
		HP	42	4.26	2.812	0.434	3.39	5.14
		Total	58	4.02	2.639	0.347	3.32	4.71
2	PCS	NHWP	16	2.44	3.521	0.88	0.56	4.31
		HP	42	8.4	9.136	1.41	5.56	11.25
		Total	58	6.76	8.399	1.103	4.55	8.97
2	WOMAC	NHWP	16	24.75	16.482	4.121	15.97	33.53
		HP	42	30.02	18.122	2.796	24.38	35.67
		Total	58	28.57	17.702	2.324	23.91	33.22
3	VAS	NHWP	5	2.2	1.924	0.86	−0.19	4.59
		HP	16	6.31	2.442	0.61	5.01	7.61
		Total	21	5.33	2.904	0.634	4.01	6.66
3	PCS	NHWP	5	4	6.364	2.846	−3.9	11.9
		HP	16	15.5	12.796	3.199	8.68	22.32
		Total	21	12.76	12.494	2.726	7.07	18.45
3	WOMAC	NHWP	5	24.4	9.45	4.226	12.67	36.13
		HP	16	40.94	20.349	5.087	30.9	51.78
		Total	21	37	19.506	4.257	28.12	45.88
4	VAS	NHWP	6	4	2.53	1.033	1.35	6.65
		HP	14	5.36	3.128	0.836	3.55	7.16
		Total	20	4.95	2.964	0.663	3.56	6.34
4	PCS	NHWP	6	6.33	5.68	2.319	0.37	12.29
		HP	14	10.79	11.477	3.067	4.16	17.41
		Total	20	9.45	10.149	2.269	4.7	14.2
4	WOMAC	NHWP	6	19.67	15.718	6.417	3.17	36.16
		HP	14	35.21	18.377	4.912	24.6	45.82
		Total	20	30.55	18.696	4.18	21.8	39.3

In terms of secondary demographic characteristics, height (STE = .577, CI = 95% [66.56–68.88], p = .006, t(193)= −2.752*,* d = −.451), Age (STE = 1.521, CI = 95% [57.12–63.24], p = .028, t(193) = −2.218, d = −.364), and NSES (STE = 2.33, CI = 95% [59.09–68.45], p < .001, t(193) = −3.684, d = −.604) in **NHWP** were all significantly greater when compared to Height (STE = .328, CI = 95% [65.28–66.57]), Age (STE = .920, CI = 95% [54.36–58.00]), and NSES (STE = 1.11, CI = 95% [53.03–57.39]) in **HP** (Table 1). Otherwise, BMI was less (p = .048, t(193) = 1.993, d = .327) in **NHWP** (STE = .783, CI = 95% [26.25–29.40]) when compared to **HP** (STE = .533, CI = 95% [28.80–30.91]). There was no difference in CCI (p = .450, t(193) = −.757, d = −.124) between **NWHP** (STE = .218, CI = 95% [1.50–2.38]) and **HP** (STE = .137, CI = 95% [1.47–2.01]). Subjects with tricompartmental disease had significantly lower VAS than Patellofemoral (p = .002) or Patellofemoral + Medial (p = .001) locations. Controlling for location, VAS (p = .005), PCS (p = .030), and WOMAC (p = .033) remained greater in **HP** compared to **NHWP**. Ethnicity*K-L grade yields no difference in WOMAC (p = 0.345), PCS (p = 0.292), or VAS (p = 0.322). Stratifying primary outcomes with ethnicity withNSES analyzed as a covariate demonstrated a similar difference in PCS (p = 0.008) and VAS (p = 0.003) as reported previously, however in this condition WOMAC is no longer different between **NHWP** and **HP** (p = 0.066). When K-L grade is tested as a covariate, WOMAC (p = 0.016), PCS (p = 0.003), and VAS (p < 0.001) remain significantly greater in **HP**.

### Multivariable models

General linear models were computed with Ethnicity as the primary independent variable and NSES and K-L grade included as covariates. These models confirmed that HP had significantly greater PCS (p = 0.005), WOMAC (p = 0.033), and VAS scores (p < 0.001) than NHWP after adjusting for both NSES and K-L grade.

### Outcomes by sex

VAS (p = .031) and WOMAC (p = .008) was greater in females when compared to males. Mean VAS in females was 4.27 (STE = .277, CI = 95% [3.73–4.82]) while in males it was 3.42 (STE = .278, CI = 95% [2.86–3.97]). Mean WOMAC in females was 29.28 (STE = 1.694, CI = 95% [25.92–32.64]) and in males was 22.63 (STE = 1.824, CI = 95% [19.00–26.25]). Neither PCS (p = .436) nor K-L grade (p = .737) was significantly different between sexes. However, mean PCS was greater in females (x̄ = 8.25, STE = .906, CI = 95% [6.46–10.05]) when compared to males (x̄ = 7.22, STE = .955, CI = 95% [5.33–9.12]). Analysis of means by Sex*Ethnicity yields no significant difference between WOMAC (.886), PCS (.393), nor VAS (p = .930).

Stratifying by sex and comparing means of primary outcomes was performed (**Table 4**). In males, **HP** (x̄ = 3.83, STE = .352, CI = 95% [3.13–4.53]) had greater mean VAS (p = .021) than **NHWP** (x̄ = 2.42, STE = .360, CI = 95% [1.68–3.16]). Male **HP** (x̄ = 8.83, STE = 1.260, CI = 95% [6.31–11.34]) had greater PCS (p = .008) than **NHWP** (x̄ = 3.35, STE = .776, CI = 95% [1.75–4.95]). Male **HP** (x̄ = 24.38, STE = 2.265, CI = 95% [19.85–28.91]) had nonsignificantly greater WOMAC (p = .136) than **NHWP** (x̄ = 18.38, STE = 2.870, CI = 95% [12.47–24.30]). Female **HP** (x̄ = 4.62, STE = .315, CI = 95% [4.00–5.25]) had greater mean VAS (p = .019) than female **NHWP** (x̄ = 3.08, STE = .521, CI = 95% [2.01–4.16]). In females, there was no significant difference between **HP** and **NHWP** in PCS (p = .164) or WOMAC (p = .177); the mean and median for both were not significantly greater in **HP** females compared to **NHWP** females. Median comparisons yield the same results, in which median VAS in females are different between **HP** and **NHWP** while median PCS and WOMAC scores in **HP** females do not significantly differ from **NHWP**.

**Table 4:** Comparison of outcomes VAS, PCS, WOMAC between HP and NHWP, stratified by sex.

							95% Confidence Interval for Mean		
Sex	Measure	Ethnicity	N	Mean	Std. Deviation	Std. Error	Lower Bound	Upper Bound	Max
Male	VAS	NHWP	26	2.42	1.837	0.36	1.68	3.16	6
		HP	63	3.83	2.791	0.352	3.12	4.53	10
		Total	89	3.42	2.619	0.278	2.86	3.97	10
	PCS	NHWP	26	3.35	3.959	0.776	1.75	4.95	15
		HP	63	8.83	10.002	1.26	6.31	11.34	39
		Total	89	7.22	9.012	0.955	5.33	9.12	39
	WOMAC	NHWP	26	18.38	14.634	2.87	12.47	24.3	59
		HP	63	24.38	17.98	2.265	19.85	28.91	85
		Total	89	22.63	17.208	1.824	19	26.25	85
	Kellgren and Lawrence Grade	NHWP	26	1.88	1.243	0.244	1.38	2.39	4
		HP	63	1.56	1.133	0.143	1.27	1.84	4
		Total	89	1.65	1.169	0.124	1.41	1.9	4
Female	VAS	NHWP	24	3.08	2.552	0.521	2.01	4.16	8
		HP	82	4.62	2.849	0.315	4	5.25	10
		Total	106	4.27	2.847	0.277	3.73	4.82	10
	PCS	NHWP	24	5.92	7.655	1.563	2.68	9.15	28
		HP	82	8.94	9.7	1.071	6.81	11.07	42
		Total	106	8.25	9.329	0.906	6.46	10.05	42
	WOMAC	NHWP	24	25.04	12.352	2.521	19.83	30.26	46
		HP	82	30.52	18.545	2.048	26.45	34.6	74
		Total	106	29.28	17.437	1.694	25.92	32.64	74
	Kellgren and Lawrence Grade	NHWP	24	1.63	1.096	0.224	1.16	2.09	4
		HP	82	1.82	1.166	0.129	1.56	2.09	4
		Total	106	1.71	1.146	0.111	1.49	1.93	4

### Outcomes by NSES, CCI, height, BMI, age, and KOA location

**Fig 2:**
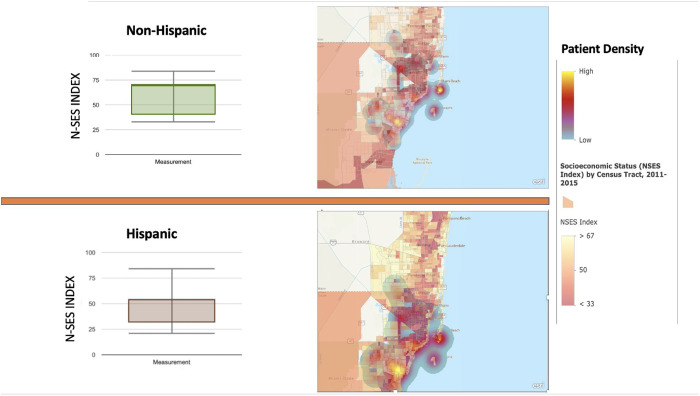
Box and Whisker plot of Neighborhood Socioeconomic Status (NSES) Index, adjacent are heat maps representative of participant address location overlayed on block-distributions for Miami-Dade County, Florida.

Comparison of socioeconomic status (SES) by group ethnicity demonstrated a statistically significant difference between the normalized socio-economic status index of **HP** and **NHWP** (p = < 0.0001) non-Hispanics more commonly resided in more affluent communities, had greater income and reported greater values in the remaining 8 parameters accounted for in the NSES index. On average, non-Hispanics on average had an NSES value of 66.9 + /- 12.4 while Hispanics resided in areas with an average NSES value of 55.5 + /- 13.6 (**Fig 2**).

Despite these findings, neither NSES (p = 0.091), CCI (p = 0.052), Height(p = 0.173), BMI (p = 0.474), or Age (p = 0.537), had any significant relationship with VAS. BMI demonstrated a direct linear relationship with PCS (p = 0.012), with a R^2^ value of 0.038 and an unstandardized beta value of 0.276. NSES (p = 0.676), CCI (p = 0.849), Height(p = 0.228), and Age (p = 0.977), showed no relationship with PCS. BMI demonstrated a positive relationship with WOMAC as well (p < 0.001), with a R^2^ value of 0.058 and an unstandardized beta value of 0.676. None of NSES (p = 0.382), CCI (p = 0.895), Height(p = 0.0.59), nor Age (p = 0.111), had a relationship with WOMAC. Both Age (p < 0.001, R^2^ = 0.207, B = 0.045) and BMI (p < 0.001, R^2^ = 0.052, B = 0.045) were positively associated with K-L grade. R^2^ value 0.207 for Age, and 0.052 for BMI. NSES demonstrated a negative association with VAS (p = 0.028, R^2^ = 0.025, B = −0.03), but no significant relationship with PCS (p = 0.190), WOMAC (p = 0.116), or K-L grade (p = 0.353). When NSES is modeled with VAS and stratified by ethnicity, the significant linear relationship no longer exists (**Table 5**).

**Table 5:** ANOVA testing evaluating the effect of NSES on VAS between HP and NHWP.

Ethnicity	Source	Sum of Squares	df	Mean Square	F	Sig.
NHWP	Regression	1.781	1	1.781	0.359	0.552
	Residual	237.839	48	4.955		
	Total	239.62	49			
HP	Regression	13.841	1	13.841	1.722	0.191
	Residual	1149.125	143	8.036		
	Total	1162.966	144			

In the **HP** group, there was a comparable prevalence of tricompartmental, medial, and bicompartmental (medial and patellofemoral) OA with percentages of 37%, 31%, and 25% respectively. For the **NHWP** group, 64% of subjects’ radiographs demonstrated tricompartmental OA, 24% depicted medial compartment OA and 7% were bicompartmental (medial and patellofemoral) (**Table 6**).

**Table 6:** Experimental data of Hispanic and Non-Hispanic White subjects included in the study. Measurements are reported as % (n).

		Ethnicity	
		NHWP	HP
KOA Location		NHWP N	NHWP Percent
Medial	N=	2	9
	Percent	4.0%	6.2%
Patellofemoral	N=	12	45
	Percent	24.0%	31.0%
Patellofemoral + Medial	N=	4	37
	Percent	8.0%	25.5%
Tricompartmental	N=	32	54
	Percent	64.0%	37.2%
Total	N=	50	145
	Percent	100.0%	100.0%

## Discussion

In the present study, we recruited three times as many **HP** compared to **NHWP** with KOA. Therefore, our sample accurately reflects the ethnicity distribution of people in southeastern Florida and builds upon recent studies that, perhaps for the first time, begin to elucidate the burden of KOA in **HP** [52,53]. Our findings that **HP** reported greater VAS, PCS and WOMAC scores compared to **NHWP** supports our first hypothesis. Perhaps more importantly, the differences between these two groups persisted after controlling for both socioeconomic status and radiographic KOA severity, further highlighting the clinical significance of ethnicity in caring for patients with this chronic disease. Our findings add new insight into the impact of NSES and radiographic grading in Hispanics with KOA, thereby contributing to the growing literature on ethnic differences in the overall burden of KOA.

Previous studies have shown that **HP** experience higher levels of pain and functional limitations than **NHWP**. A recent investigation demonstrated that **HP** reported higher pain and symptom scores despite fewer changes on plain radiographs compared with **NHWP** with KOA [52]. Interestingly, a recent review by Hollingshead et al concluded that **HP** report more severe chronic pain, chronic joint pain, average chronic pain over the past week, and fewer pain free days in national surveys [26]. Our observations build upon these studies by showing that **HP** with KOA continue to report higher levels of pain in surveys when potential confounding variables including NSES and radiographic grading are accounted for, thereby suggested a true need for tailored care.

It has also been shown that Non-Hispanic Black patients similarly report greater values for PCS, VAS, and WOMAC in comparison to **NHWP** with KOA [25,53–59]. The *Dunlop et al*. study noted non-Hispanic black and Hispanic older adults reported having symptomatic arthritis at a substantially higher frequency than did non-Hispanic whites [60]. Fabian et al. explored these cultural differences and found that catastrophizing varied by ethnicity, with African Americans reporting greater catastrophizing than Asian/Pacific Islanders and Caucasians [61]. The authors also found that situational catastrophizing significantly mediated pain intensity [61]. Similarly, Bishop et al. associated intensification of pain and a greater level of pain catastrophizing for individuals with chronic low back pain [49]. Dagenias et al. conducted a systematic review for acute lower back pain and arrived at the same conclusion; PCS and pain intensity were positively associated [62]. Furthermore, Miller et al. noted that pain catastrophizing is positively associated with pain experience and functional limitation [63]. Moreover, this association is influenced by ethnicity even after accounting for social economic status (SES) and disease severity [63].

We also evaluated NSES as a possible mediator of pain, pain catastrophizing, and functional limitation in our patients with KOA. Interestingly, we identified that Hispanics tended to be more concentrated in lower NSES communities that were situated more inland within Miami-Dade County, Fla. Non-Hispanic participants tended to reside closer to the county coastline and tended to have greater NSES status. Yet, after pairing individuals based on NSES value, **HP** reported greater pain, pain catastrophizing, and functional limitations. These differences can likely be explained in part by two prior investigations using the Catastrophizing subscale of the CSQ* that found individuals of Hispanic ethnicity with chronic pain used catastrophizing as a coping method more than **NHWP** [55,63]. Moreover, we did not analyze the relationships between NSES and PCSs within group therefore a statement of NSES and PCS correlation would be inappropriate. Nevertheless, work by Feldman and others have noted that lower NSES is usually associated with greater PCS scores possibly due to reduced access to healthcare, inadequate treatment, or specific occupational hazards [34].

As K-L grade increased in our patient sample, a worsening VAS, PCS, and WOMAC trend was seen in both **NHWP** and **HP**, though this trend was only significant for each outcome in **HP**. Of note, **HP** had a greater prevalence of OA in the medial and patellofemoral compartments when compared with **NHWP**. We propose that this difference in anatomic distribution of OA among the 2 groups may help to account for the differences in VAS [55]. Further study is needed however to confirm our theory and how that may potentially affect treatment and outcome.

The findings of our study may be further explained by theories not tested in our study. Cultural aspects of **HPs** may play a significant role in the experience of pain. Research has shown that children are more likely to develop chronic pain if their parent or sibling also experiences chronic pain [64]. Therefore, a patient’s experience of chronic pain may be influenced by their kin in a cycle that continues to propagate values higher than **NHWP**. Additionally, **HP** tend to work in occupations that expose them to greater levels of safety risk [65], which may play a role in higher reporting to these surveys. Future investigations are required to isolate these variables due to the multifactorial nature of the pain experience in order to further understand why **HP** report higher than **NHWP** to these surveys [66]. The findings of our study may also influence future studies done in predominantly Hispanic regions to take ethnicity into account when evaluating pain experience or aid providers in coordinating a unique treatment plan based on patient ethnicity.

As with any study, there are limitations. First, our data collection was carried out at a tertiary clinic, making our patient population biased to those who can access our healthcare center including availability, transportation, and insurance. Second, when utilizing NSES data we inferred individual NSES based on neighborhood values which are an approximation and do not report the individual’s level of education, type of occupation nor individual disposable income among other characteristics. Accordingly, this measurement may be subject to error. In the same vein, our analysis was restricted to Miami-Dade County and failed to include 9 individual **NHWP** who lived in Broward County and 1 individual who lived in Colorado. Given the limited sample size in the sub analysis (n = 40), the inclusion of these individuals would likely have influenced study results. Moreover, we used a cross-sectional study design where our evaluation of pain was at single time point that is likely not representative of the individual patient’s overall pain experience. Our study however has several strengths. Participant sampling was conducted in Miami-Dade County, Florida, with a large diaspora of the Hispanic population providing greater access to this minority population and to our knowledge the largest sample size to date of this minority group. Our data collection tools have been validated for Hispanic persons and were administered in a culturally competent manner. Finally, our GIS application for NSES has been previously utilized as a measure for NSES in various populations, including Hispanic persons [34].

## Conclusion

Disparities in the treatment of patients with OA involving different racial, ethnic, and socioeconomic groups are well described [67]. We examined the pain experience of **HP** and **NHWP** presenting with symptomatic KOA and demonstrated that differences between the 2 groups are significant for VAS, PCS and WOMAC, which persisted after controlling for NSES and K-L grade. This observation highlights the impact of both K-L grade and NSES on pain, function, and pain catastrophizing coping for individuals with KOA. Given that the treatment of individuals with OA is based largely on patient reported symptoms, our findings suggest including a PCS inventory that provides information to the expected severity pain experience may be of value in the assessment and treatment of Hispanic patients.
